# Electroacupuncture Attenuates Anxiety-Like Behaviors in a Rat Model of Post-traumatic Stress Disorder: The Role of the Ventromedial Prefrontal Cortex

**DOI:** 10.3389/fnins.2021.690159

**Published:** 2021-06-24

**Authors:** Yuchao Hou, Meiyu Chen, Can Wang, Lumin Liu, Huijuan Mao, Xiaoyi Qu, Xueyong Shen, Bo Yu, Sheng Liu

**Affiliations:** ^1^Department of Acupuncture-Moxibustion and Tuina, Shanghai University of Traditional Chinese Medicine, Shanghai, China; ^2^Department of Human Anatomy, School of Basic Medicine Sciences, Shanghai University of Traditional Chinese Medicine, Shanghai, China

**Keywords:** posttraumatic stress disorder, electroacupuncture, anxiety-like behaviors, ventromedial prefrontal cortex, ventral tegmental area

## Abstract

Electroacupuncture (EA) is a promising clinical approach to treating posttraumatic stress disorder (PTSD), yet the mechanisms whereby EA can alleviate anxiety and other PTSD symptoms have yet to be clarified. In the present report, rats underwent EA for 14 consecutive days following modified single prolonged stress (MSPS) exposure. These animals were then evaluated in open field and elevated plus maze tests (OFT and EPM), while Fos immunohistochemical staining was performed to assess ventromedial prefrontal cortex (vmPFC) functional activation. In addition, an extracellular recording and stimulation system was used to analyze vmPFC inputs into the ventral tegmental area (VTA) in these rats. Temporary vmPFC inactivation was further performed to assess whether this was sufficient to reverse the anxiolytic effects of EA. Overall, rats that underwent EA treatment spent more time in the central region (OFT) and the open arm (EPM) relative to MSPS model animals (*P* < 0.05). These MSPS model animals also exhibited significantly fewer activated Fos-positive nuclei in the vmPFC following behavioral testing, while EA was associated with a significant relative increase in c-Fos expression in this region. The transient inactivation of the vmPFC was sufficient to reverse the effects of EA treatment on anxiety-like behaviors in MSPS model rats. MSPS and SEA rats exhibiting no differences in bursting activity between baseline and vmPFC stimulation, whereas bursting activity rose relative to baseline upon ventral mPFC stimulation in EA treated and control rats. Together, these findings indicate that the vmPFC and its inputs into the VTA are functionally linked to the anxiolytic activity of EA, implicating this pathway in the EA-mediated treatment of PTSD.

## Introduction

Posttraumatic stress disorder (PTSD) is a serious psychological condition that can arise in individuals who have experienced or witnessed a traumatic event, causing a range of symptoms including depression and anxiety that can adversely impact an affected individual’s overall quality of life (Woodward et al., 2000). Both pharmaceutical- and psychotherapy-based approaches have been used to treat the symptoms of PTSD (Taylor et al., 2006). Acupuncture is a traditional Chinese medicinal practice that has been conducted for over 2,000 years, and that can alleviate stress-related anxiety and depression *via* the hypothalamic-pituitary-adrenal axis (HPA) (Grant et al., 2018; Oh et al., 2018). Hollifield et al. (2007) determined that PTSD patients undergoing acupuncture exhibited significant decreases in clinical symptoms comparable to those of patients undergoing cognitive-behavioral therapy. Experiments from our lab and others have shown that electroacupuncture (EA) can achieve anxiolytic activity in PTSD model rats (Oh et al., 2018; Li et al., 2019; Liu et al., 2019; Xue et al., 2019). The mechanisms whereby this form of acupuncture can treat anxiety-related behaviors associated with PTSD, however, remain to be fully clarified.

The ventromedial prefrontal cortex (vmPFC) is located in the medial prefrontal cortex, and plays central roles in regulating both goal-directed and anxiety-related behaviors (Kim et al., 2011; Arnsten et al., 2012; Padilla-Coreano et al., 2016). The pathophysiological basis of PTSD is thought to be partially attributable to reductions in vmPFC top-down emotional modulation (Nicholson et al., 2017). Abnormal apoptotic cell death within the vmPFC has recently been detected in the traditional single prolonged stress (SPS) model of PTSD (Jia et al., 2018; Pati et al., 2018), and SPS model animals also exhibit reductions in vmPFC c-Fos immunoreactivity (Yu et al., 2015; Pati et al., 2018). Furthermore, SPS can induce hypoactivity in the vmPFC and impaired prefrontal cortex control of amygdala and striatum in rats (Piggott et al., 2019). There is also a growing body of evidence suggesting that anxiety modulation and PTSD incidence are related to the dopamine (DA) system in the ventral tegmental area (VTA) (Corral-Frias et al., 2013; DeGroot et al., 2020). The inactivation of DA neurons within the VTA can reduce PTSD-like behavior incidence or intensity, in addition to significantly reducing baseline firing of these dopaminergic VTA cells (Corral-Frias et al., 2013). Furthermore, vmPFC inputs into the VTA play a key role in the functionality of this DA system and the associated regulation of anxiety (Knowland and Lim, 2018), with the vmPFC exhibiting direct innervation of DA neurons in the VTA (Gariano and Groves, 1988), controlling DA release within the nucleus accumbens (Taber et al., 1995; You et al., 1998). Some researchers have posited that vmPFC afferents to the VTA may serve as a key source of anxiety-related glutamate within the VTA (Felix-Ortiz et al., 2016). Recent studies have shown that acupuncture involves cortical modulation (Hauck et al., 2017). The anterior cingulate cortex, for example, is critical for the effects of EA on anxiety-associated behaviors in both SPS and formalin-induced pain rat model systems (Yi et al., 2011; Liu et al., 2019). Prior work from our lab has also shown that EA can alter vmPFC neuron firing activity (Zhang et al., 2017).

Herein, we utilized a rat SPS model system to explore the impact of EA on anxiety-like behaviors through its ability to influence vmPFC neurons and inputs into the VTA. Functional vmPFC activation following EA was assessed *via* Fos immunomapping, while behavioral experiments were used to assess anxiety-related behaviors. Temporary vmPFC inactivation was additionally conducted to confirm whether such inactivation was sufficient to reverse the anxiolytic effects of EA. In addition, extracellular recordings of anesthetized rats were employed to investigate whether PTSD was associated with vmPFC inputs into the VTA and whether these effects were reversed upon EA treatment.

## Materials and Methods

### Animals

Male Sprague-Dawley (SD) rats (220–250 g) from the Shanghai Experimental Animal Center were purchased and allowed to acclimate for 7 days prior to experimental use. Rats were housed in a controlled environment (25 ± 2°C, 12 h light/dark cycle) with free food and water access. This study was conducted as per NIH standard guidelines and received approval from the Shanghai University of Traditional Chinese Medicine Animal Care and Use Committee.

### Modified Single Prolonged Stress

An MSPS model was established as reported previously (Su et al., 2013; Perrine et al., 2016), with modifications having been made in our laboratory (Liu et al., 2019). Briefly, rats were subjected to three consecutive stressors: restraint, forced swim, and anesthesia stresses. Restraint was achieved by placing rats into plexiglass cylinders for 2 h such that they experienced head immobilization. Rats were then placed in an acrylic cylindrical bucket (40 cm diameter, 50 cm height) that contained fresh water (20–25°C, two-thirds full) and forced to swim for 20 min. After drying and recovering for 15 min, rats were anesthetized to unconsciousness such that no response to tail or toe pinch was evident using pentobarbital sodium. Rats were then returned to their cages for 7 days.

### EA Treatment

Electroacupuncture stimulation was conducted while rats were gently restrained. Sterile stainless steel needles (0.16 mm diameter, 13 mm long) were inserted bilaterally into acupoint ST36 between the anterior tibialis and extensor digitorum longus muscles proximal to the knee joint. Electric stimulation was generated by a stimulator instrument (Shanghai Medical Electronic Apparatus, China), and was delivered using two needles. The frequency of stimulation was 2 Hz. The intensity of the stimulation was increased stepwise from 0.5, 1.0 to 1.5 mA, with each step lasting for 10 min. Sham control rats had needles inserted into these ST36 acupoints but did not undergo electrical stimulation.

### Behavioral Testing

#### Elevated Plus Maze Testing

An EPM apparatus (Shanghai Jiliang Software Technology Co., Ltd.) was used for this study. This maze was composed of two open arms facing in opposite directions and two closed arms facing in opposite directions that were 50 cm above the floor (all arms were 15 cm wide and 45 cm in diameter). As detailed previously (Liu et al., 2019), rats were placed in the center of the maze facing an open arm at the start of the test, and were allowed to roam freely for 15 min during which time a video tracking apparatus recorded their movement and behaviors with the EthoVision software (v7.1). Both the time spent in open arms and the percentage of time spent in open arms were measured as exploratory behaviors of interest, with an arm entry being recorded when a rat entered a given maze arm with all four paws.

#### Open Field Test Analyses

Open field test analyses are routinely used when measuring anxiety and spontaneous locomotor activity (Schmitt and Hiemke, 1998). Briefly, rats were placed in the central region of 40 cm × 40 cm × 50 cm apparatus that was separated into central and peripheral regions by gray lines. Rats were then allowed to freely roam through this apparatus for 15 min, during which time a video-tracking system (Shanghai XinRuan Information Technology Co., Ltd.) recorded their behavior, with the EthoVision software [v 7.1] being used to automatically analyze the time spent in the central region and the total distance covered by these rats.

### c-Fos Immunohistochemical Staining

At 1.5 h post-behavioral testing, rats were deeply anesthetized using pentobarbital sodium (100 mg/kg, ip), sequentially perfused transcardially with saline (200 mL) and 4% paraformaldehyde (PFA) in 0.1 mol/L phosphate buffer (PB) (250 mL). Brains were then collected from each animal, fixed overnight in 4% PFA, and transferred to 30% sucrose for 5–7 days until the tissue was saturated and had sunk to the bottom of the solution. Samples were then sliced to yield a series of 30 μm-thick coronal sections using a chilled (−25°C) cryostat instrument, with the resultant sections being transferred to PBS. The staining of these sections for c-Fos was conducted as in prior studies (Georges et al., 2006). Briefly, sections were washed thrice with PBS, blocked for 2 h with 1% BSA at 4°C, and probed for 48 h with mouse monoclonal anti-c-Fos (1:400; sc-166940, Santa Cruz) at 4°C. Following three subsequent washes with PBS, sections were stained for 2 h with mouse IgGk light chain-binding protein (m-IgGk BP-PE, 1:200; sc-516141, Santa Cruz). Sections were then mounted on adhesive slides to which coverslips and fluorescence decay-resistant medium were applied. A Leica Laser Scanning Confocal Microscope was then used to analyze these stained sections. The best standard stereotaxic plane sections of the vmPFC were identified as per Paxinos and Watson’s atlas (George and Charles, 2013), with numbers of c-Fox positive cells per section then being assessed (20×). An automatically generated 200 μm × 500 μm rectangle was used to denote the vmPFC in each section, and an analytical software was used to calculate the number of stained nuclei per section. Numbers of c-Fos-positive nuclei per section were thereby determined and averaged to yield representative results for analysis.

### Pharmacological Inactivation

One week before EPM testing, rats (300–350 g) were intraperitoneally injected with pentobarbital sodium (50 mg/kg) and mounted on a stereotaxic frame, with isoflurane (1.5–2%) being delivered *via* a nosecone. Bilateral guide cannulae (26 gauge, Plastics One) were implanted in the vmPFC (+ 3.0 mm AP; ± 0.8 mm ML; –3.8 mm DV). The cannulae were fixed to the skull with dental cement and three steel screws. To ensure that the cannulae remained unobstructed, stainless steel obturators were inserted. In EA group, rats were cannulated after EA treatment. Rats were systemically treated with benzylpenicillin sodium (60,000 U) to prevent infection, and were allowed to recover for 5–7 days after surgery.

Five minutes prior to EPM testing, rats were bilaterally injected with 0.3 μl of artificial CSF (aCSF) or an equivalent volume of aCSF containing 1.0 nmol/0.1 nmol mixture of baclofen and muscimol (GABAB and GABAA receptor agonists, respectively; Tocris Bioscience). For these injections, 33-gauge injection cannulae were inserted into the guides and extended 1 mm below the guide cannula tip, with solutions being delivered through PE50 tubing *via* microinfusion pump over a 1 min period using a microsyringe. Following injection, cannulae were allowed to remain in place for 1 min, and were then removed to permit fluid diffusion.

Following behavioral testing, rats were euthanized *via* sodium pentobarbital overdose (100 mg/kg, ip), perfused sequentially with 0.9% saline and 4% PFA in PBS (250 mL), after which brains were collected, stored in 30% sucrose as above, frozen, and sliced with a cryostat to yield 40 μm sections. Cannula placement was confirmed by comparing cannula-related damage to reference images in a rat brain atlas (George and Charles, 2013). Due to cannula misplacement, one rat was excluded from behavioral testing analyses.

### Stimulation and Recording

#### Stereotaxic Surgery

Initial anesthetization was achieved by placing rats in a closed container containing 2.5% isoflurane, after which a tracheotomy was conducted and a 1.0–1.5% isoflurane solution was delivered under spontaneous respiration *via* a tracheal cannula to maintain anesthetization during surgery. Rats were mounted in a stereotaxic apparatus and warmed to 36–38°C with a heating pad. The skull was then exposed, and holes above the vmPFC (2.8 mm rostral and 0.4–0.6 mm lateral to bregma) and the VTA (5.3 mm caudal and 0.5–0.8 lateral to bregma) were drilled.

#### Electrical Stimulation of the vmPFC

A bipolar concentric electrode (250 μm in diameter overall; 50 μm diameter for inner electrode) was inserted into the vmPFC (5.0 DV from skull surface). Electrical stimulation was then achieved using a square pulse stimulator controlled by the Spike2 program (CED 1401, Spike2; Cambridge Electronic Design, Cambridge, United Kingdom) (0.5 Hz, 0.5 ms pulse duration). Two intensity levels (0.5 and 1.0 mA) were tested during stimulation.

#### VTA Recording

Recording in the VTA was achieved by inserting a glass micropipette (tip diameter, 1–3 μm; 6–12 MΩ) containing a solution of 2.0% pontamine sky blue solution in 0.5 M sodium acetate into the VTA (DV: 6.5–9.0 mm). Electrophysiological criteria detailed previously were used to identify spontaneously active DA neurons in this region (Tong et al., 1996; Georges et al., 2006; Ungless and Grace, 2012). DA neuron activities recorded within the VTA included: (1) action potentials (APs) exhibiting biphasic or triphasic waveforms > 2.5 ms in duration, (2) > 1.1 ms from spike onset to negative trough, and (3) a slow spontaneous firing rate [> 10 spikes/second (sp/s)]. Bursts were defined by the detection of two consecutive spikes with an interspike interval < 80 ms, whereas burst termination was defined by two spikes for which this interval was > 160 ms (Tong et al., 1996; Georges et al., 2006; Ungless and Grace, 2012; Kaufling and Aston-Jones, 2015). Signals were amplified and filtered (0.1–5 kHz bandpass) with standard electronic equipment. Single neuron spikes were identified and interpreted as digital pulses by the computer through the use of the Spike2 software. Upon isolating a single neuron, spontaneous baseline activity prior to stimulation was recorded for at least 5 min, after which 50 individual electrical pulses were delivered to the vmPFC, and the responses of these VTA DA neurons were recorded.

### Histological Analysis

Electrode locations were verified after recording session completion. Sites of vmPFC stimulation were demarcated by applying 10–20 μA of positive current for 1–2 min through the stimulating electrode to generate a lesion, while sites of VTA recording were demarcated based upon the presence of iontophoretic pontamine sky blue deposits following the application of an alternating current (−7 μA) for 12–15 min. Rats were then euthanized, and brain tissue sections were prepared as above with recording sites being verified by Leica Microsystems.

### Statistical Analysis

Data are means ± SEM. Behavioral and c-Fos staining data were analyzed *via* one-way ANOVAs with Fisher’s *post hoc* LSD test where appropriate. *P* < 0.05 was the threshold of significance for these analyses.

For electrophysiological analyses, both firing rate and the percentage of spikes occurring in bursts (%SIB) were analyzed. Burst onset was defined by the detection of two spikes within < 80 ms of one another (Schmitt and Hiemke, 1998), while%SIB was calculated before and after vmPFC stimulation by dividing the total number of spikes that occurred in bursts by the total spike number over the measured time period. Excitatory and inhibitory epochs of VTA DA neurons in response to stimulation of the vmPFC were assessed as detailed previously (Jodo et al., 1998; Moorman and Aston-Jones, 2010). During vmPFC stimulation, cumulative VTA activity peri-stimulus time histograms (PSTHs) with a bin width of 5 ms were generated for all recorded neurons (Jodo et al., 1998). These PSTHs were used to assess response magnitude (Rmag) for excitation and inhibition, and were normalized to baseline spontaneous firing activity levels. Briefly, baseline average counts (per bin) were initially determined over the 500 ms period prior to stimulation, with excitation onset being the first five bins in which the mean value was greater than two standard deviations above mean baseline activity levels. Response offset was defined as the time when this activity had returned to levels within two standard deviations of the baseline mean values. Inhibition was defined as the presence of an epoch at least 15 bins in length during which the mean count per bin was at least 35% below baseline. The resultant data were given as means ± SEM, and were analyzed *via* one-way ANOVAs with Fisher’s LSD test as appropriate. Chi-squared tests were used to compare relative proportions of inhibited or excited neurons in the vmPFC between treatment groups, with mean firing rate and %SIB values during vmPFC stimulation within each group were compared using paired *t*-tests.

## Results

### EA Treatment Impacts MSPS-Related Anxiety-Like Behaviors

We began by using the OFT and EPM tests to evaluate the impact of EA treatment on anxiety-like behaviors in MSPS model rats. OFT and EPM test were conducted in the morning and afternoon, respectively, with at least 4 h between tests to ensure they did not interfere with one another. Rats were randomized into four groups (*n* = 8/group): control, MSPS, EA, and Sham EA (SEA) groups. MSPS group rats did not undergo EA, while control group rats did not undergo MSPS modeling. EA group rats were treated for 30 min/day for 14 consecutive days beginning 1 week after MSPS model establishment, while SEA rats underwent the same treatment regimen but without the application of electrical stimulation during EA therapy.

### EA Alters MSPS Model Rat Performance in OFT Analyses

**FIGURE 1:**
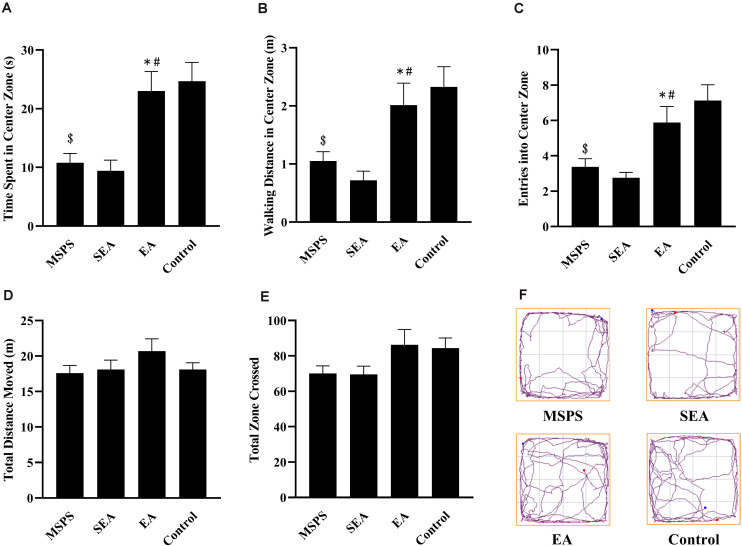
EA at ST36 alters MSPS model rat anxiety-like behaviors in OFT experiments. **(A)** Time spent in the central region in seconds. **(B)** Distance traveled in the central region (m). **(C)** Central region entries. **(D)** Total distance moved (m). **(E)** Total zone crossings. **(F)** Representative tracking plots. Data are means ± S.E.M. (^$^*P* < 0.05, PTSD vs. control groups; *P < 0.05, EA vs. SEA groups; ^#^*P* < 0.05, EA vs. PTSD groups).

In OFT analyses, significant differences were detected among rats in the four different treatment groups with respect to the amount of time spent in the central region [*F*(3,28) = 9.337, *P* < 0.001], the distance traveled in the central region [*F*(3,28) = 7.49, *P* < 0.001], and entries into the central region [*F*(3,28) = 8.753, *P* < 0.001] (Figure 1). Through *post hoc* analyses, we determined that rats in the MSPS group exhibited significant reductions in time spent in the central region (*P* < 0.001), distance traveled in the central region (*P* < 0.01), and entries into the central region (*P* < 0.001) relative to control rats. Relative to rats in the MSPS model group, EA group rats spent significantly more time in the central region (*P* < 0.01), in addition to exhibiting increased walking distance in the central region (*P* < 0.05), and more entries into the central region (*P* < 0.05). No such difference, in contrast, was observed for rats in the SEA group relative to the MSPS group. No differences were observed among groups with respect to zones crossed [*F*(3,28) = 2.175, *P* > 0.05] or total distance traveled [*F*(3,28) = 1.163, *P* > 0.05].

### EA Alters MSPS Model Rat Performance During EPM Testing

**FIGURE 2:**
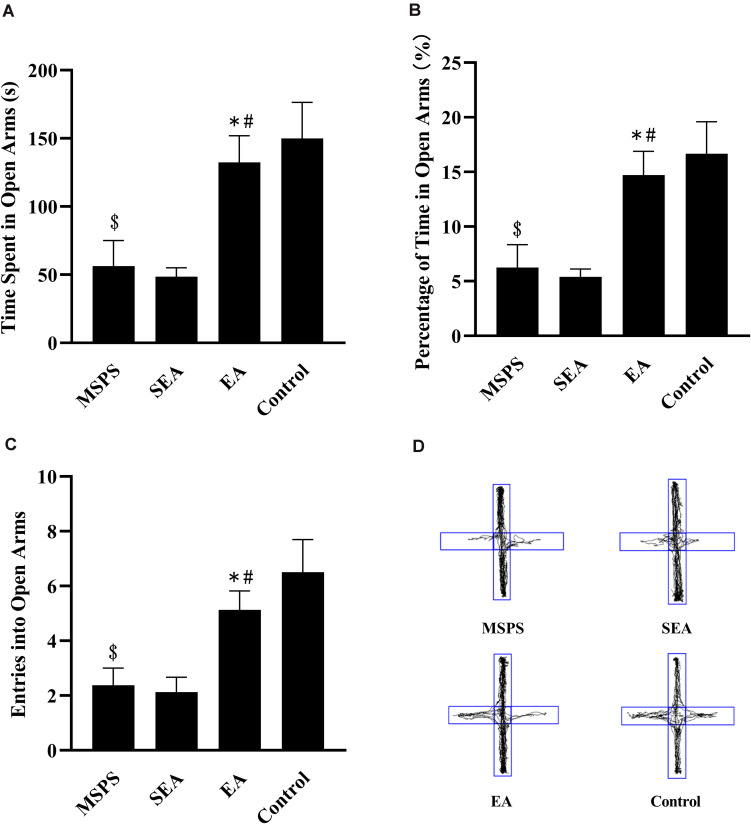
Impacts of EA at ST36 on anxiety-like behaviors in MSPS rats as measured in an EPM. **(A)** Time spent in the open arms in seconds. **(B)** Percentage of time spent in open arms. **(C)** Open arm entries. **(D)** Representative tracking plots. Data are means ± S.E.M. (^$^*P* < 0.05, PTSD vs. control groups; *P < 0.05, EA vs. SEA groups; #*P* < 0.05, EA vs. PTSD groups).

Significant differences were detected *via* one-way ANOVA when comparing rats in the four treatment groups with respect to time spent in the open arms [*F*(3,28) = 7.316, *P* < 0.001], percentage of time spent in open arms [*F*(3,28) = 7.317, *P* < 0.001], and open arm entries [*F*(3,28) = 7.008, *P* < 0.001]. Rats in the MSPS group were less active in open arms than were control rats (Figure 2D), exhibiting significant decreases in the amount of time spent in open arms (*P* < 0.01; Figure 2A), the percentage of time spent in open arms (*P* < 0.01; Figure 2B), and open arm entries (*P* < 0.01; Figure 2C). Relative to these MSPS model animals, those in the EA group exhibited significant increases in time spent in open arms (*P* < 0.01), open arm entries (*P* < 0.05), and percentage of time spent in open arms (*P* < 0.01), whereas such differences were not observed when comparing rats in the MSPS and SEA groups (*P* > 0.05).

### EA Treatment Alters vmPFC c-Fos Expression Following Behavioral Testing in MSPS Model Rats

**FIGURE 3:**
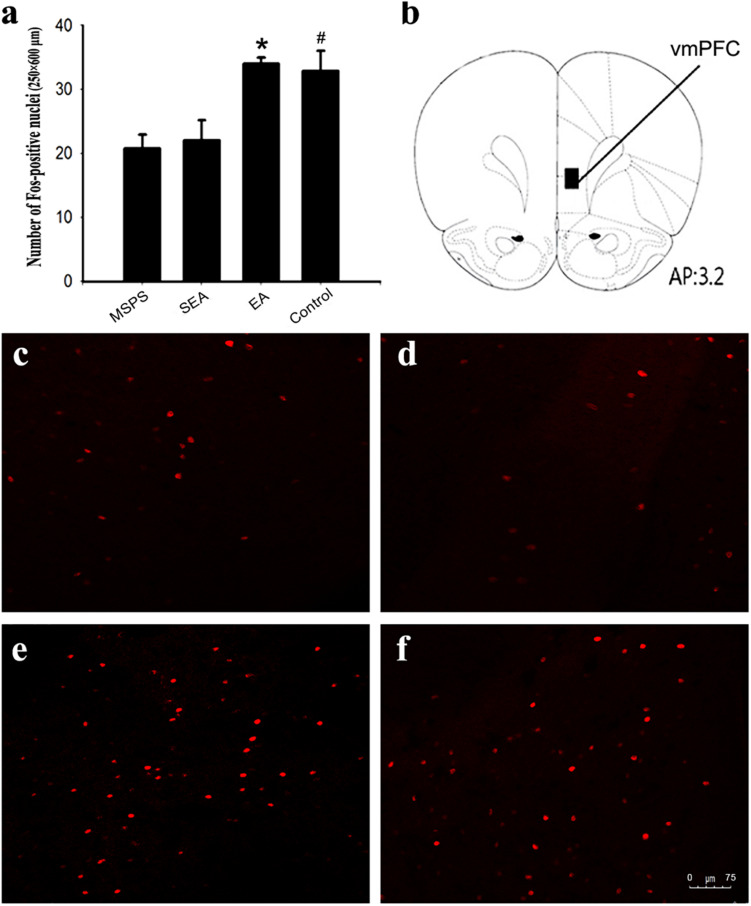
EA treatment increases the number of Fos-positive nuclei in the vmPFC of MSPS model rats. **(a)** Quantitative assessment of Fos-positive nuclei in the vmPFC. Data are means ± SEM. *#*P* < 0.05 vs. PTSD group. **(b)** Schematic overview of a coronal section through the vmPFC, with a 200 μm × 500 μm rectangle highlighting the region of the vmPFC in which Fos-positive nuclei were counted. The number at the bottom of the schematic indicates the distance from the bregma in millimeters. This image was adapted from an atlas produced by Paxinos and Watson. **(c–f)** Representative coronal sections illustrating Fos staining in the vmPFC: **(c)** MSPS group; **(d)** SEA group; **(e)** EA group; **(f)** Control group.

The pathophysiological basis for PTSD is thought to be at least partially attributable to reduced top-down emotion modulation from vmPFC regions (Nicholson et al., 2017). To examine the link between the beneficial effects of EA on anxiety-like behaviors and vmPFC functional activation, c-Fos immunomapping was thus conducted. At 90 min post-behavioral testing, four rats per group were selected at random. Three consecutive sections were taken from each animal for IHC staining analyses of the vmPFC region. Significant differences in vmPFC c-Fos levels were observed among treatment groups [*F*(3,44) = 7.19, *P* < 0.01] (Figure 3). Specifically, there were significantly fewer Fos-positive neurons in the MSPS and SEA groups relative to the control group (Figures 3a,c,d), whereas these numbers were significantly higher in rats in the EA group relative to those in the MSPS and SEA groups (*P* < 0.01, Figures 3a,e). No differences between the EA and control groups were observed with respect to c-Fos expression in the vmPFC (*P* > 0.05, Figures 3a,e,f).

### Transient vmPFC Inactivation Ablates the Effects of EA on Anxiety-Like Behaviors

**FIGURE 4:**
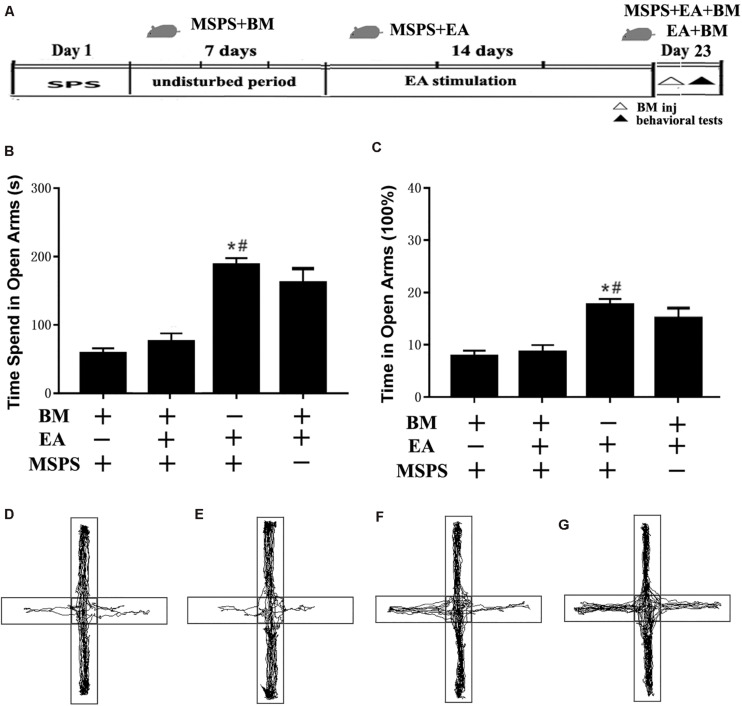
Transient vmPFC inactivation disrupts the effects of EA on anxiety-like behaviors in MSPS model rats. **(A)** A timeline of MSPS modeling, EA treatment, stereotactic injection, and behavioral testing protocols. Briefly, MSPS modeling was performed on day 1. After a 7-day rest, rats were treated once per day as per an EA protocol (days 9–22). On day 23, rats were stereotaxically injected with artificial CSF (aCSF) or a 1.0 nmol/0.1 nmol mixture of baclofen and muscimol (BM) in the bilateral vmPFC immediately prior to behavioral testing. **(B)** Time spent in the open arms during elevated plus maze (EPM) testing for rats in the indicated groups. **(C)** Percentage of time spent in open arms. **(D–G)** Real-time movement traces during EPC testing: **(D)** BM + MSPS group; **(E)** BM + MSPS + EA group; **(F)** EA + MSPS group; **(G)** EA + BM group. *#*P* < 0.05 vs. the BM + MSPS group and the BM + MSPS + EA group.

To clarify the relationship between the vmPFC and the apparent anxiolytic responses associated with EA treatment in PTSD model rats, we next conducted transient vmPFC inactivation immediately before behavioral testing to see whether this was sufficient to reverse the anxiolytic responses observed following EA treatment (Figure 4A). One-way ANOVAs indicated that such treatment was associated with significant differences in time spent in open arms [*F*(3,20) = 7.59, *P* < 0.001] and the percentage of time spent in open arms [*F*(3,20) = 8.33, *P* < 0.001] among groups, with EA treatment having augmented the amount of time spent in open arms as above (MSPS + EA vs. MSPS + BM, *P* < 0.05, Figures 4B,C,F). Prior research has indicated that inactivating the vmPFC can result in increased anxiety as measured *via* EPM testing (de Visser et al., 2011; Pati et al., 2018). We similarly found that the observed increases in time spent in open arms during EPM testing in the EA group were ablated by transient vmPFC inactivation. Indeed, animals in the MSPS + EA + BM group exhibited significantly less time spent in the open arm during EPM analyses relative to rats in the MSPS + EA group (MSPS + EA vs. MSPS + EA + BM, *P* < 0.05, Figures 4B–E,G).

### VTA Inputs From the vmPFC Are Impacted by MSPS and EA Treatment as Determined Through Electrophysiological Analyses

**FIGURE 5:**
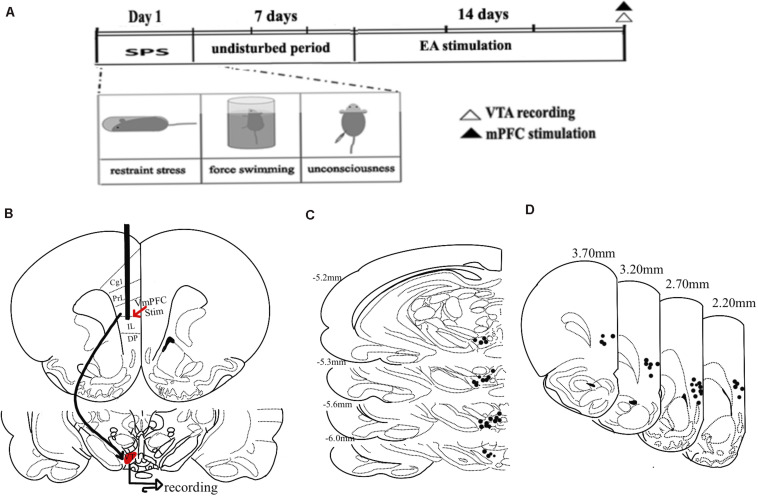
Experimental timeline overview and vmPFC stimulation and recording site schematics. **(A)** A timeline of MSPS modeling, EA treatment, electrophysiological stimulation, and recording. **(B)** An overview of the vmPFC electrophysiological stimulation and recording sites used for this study. **(C)** Plots of 55 histologically localized DA neurons within the VTA. **(D)** Hemisection plots of the prefrontal cortex indicating stimulation site distributions.

**FIGURE 6:**
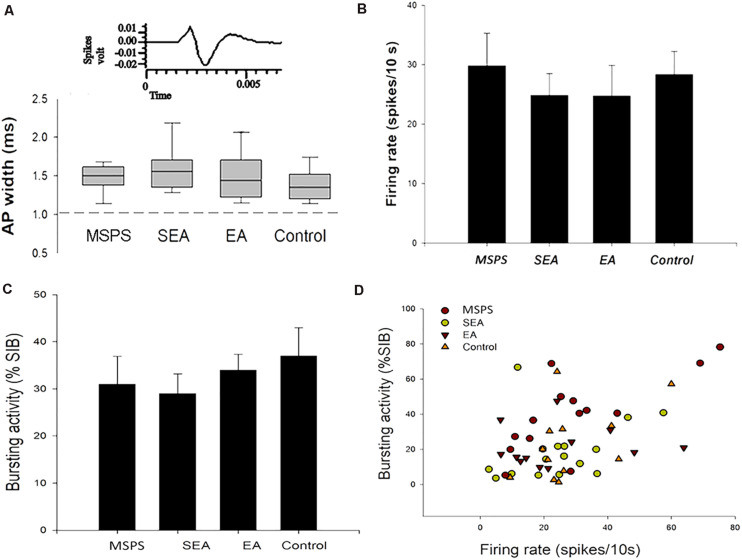
MSPS and EA treatment alter VTA DA neuron responses. **(A)** Representative average extracellular neuron waveforms. The width of an action potential (AP) was measured from the start of that potential to the apex of the negative trough, and similar normal distributions of AP widths (range: 1.1–2.7 ms) were detected in PTSD rats, SEA rats, EA rats, and controls. **(B–D)** Average firing rates and bursting activity (%SIB) of VTA DA neurons in the indicated treatment groups.

Ventral tegmental area inputs from the vmPFC play a key role in the overall function of the DA system, regulating its influence on anxiety-like behaviors following trauma (Taber et al., 1995; Arnsten et al., 2012; Corral-Frias et al., 2013). To explore VTA DA neuron responses to vmPFC electrical stimulation in the context of MSPS modeling and EA treatment, we next conducted *in vivo* extracellular single-unit recording studies of 55 histologically verified DA neurons from 25 rats based on their exhibiting wide spikes (>2.5 ms) and a wide initial action potential (AP) component (Ungless and Grace, 2012). GABA-like neurons exhibiting rapid firing rates and think spikes were not a focus of the present study. An overview of the experimental workflow for these experiments is shown in Figure 5A, with stimulation sites being shown in Figures 5B,C, and with the plots of these 55 histologically localized DA neurons recorded in the VTA being shown in Figure 5D. All DA neurons recorded in this study exhibited an AP width > 1.1 ms (Ungless et al., 2004), and no differences were observed among groups with respect to the neurons meeting these criteria (Figure 6A) [ANOVA; *F*(3,51) = 2.15; *p* > 0.05]. VTA DA neurons in PTSD group rats exhibited a mean firing rate of 29.8 ± 5.5 spikes/10 s, while these rates were 24.9 ± 3.7 spikes/10 s, 24.8 ± 5.2 spikes/10 s, and 28.4 ± 3.9 spikes/10 s in SEA, EA, and control rats, respectively (Figure 6B). The percentage of spikes occurring in bursts (%SIB) in the PTSD, SEA, EA, and control groups was 31.2 ± 5.8%, 29.1 ± 4.2%, 34.2 ± 3.3%, and 35.3 ± 5.6%, respectively (Figures 6C,D), with the number of spikes per burst in these four respective groups being 3.0 ± 0.43, 2.1 ± 0.25, 2.7 ± 0.25, and 2.4 ± 0.2. None of these variables differed significantly among these analyzed groups (*P* > 0.05).

**FIGURE 7:**
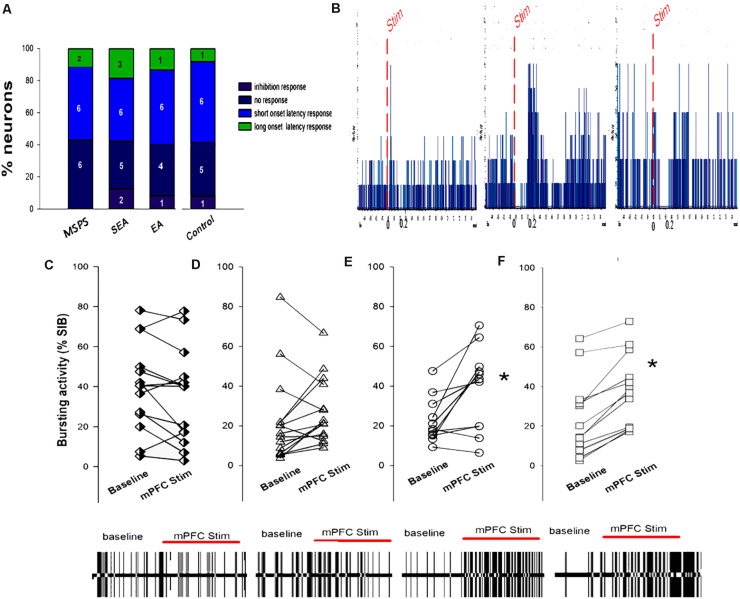
The association between MSPS and EA treatment VTA DA neuron responses to vmPFC electrical stimulation. **(A)** VTA DA neuron response types in response to vmPFC stimulation in the indicated treatment groups. **(B)** Representative DA neuron short-latency excitatory responses (left), long-latency excitatory responses (middle), and inhibition responses (right) following vmPFC stimulation (50 pulses, 0.5 Hz). **(C–F)** VTA DA neuron bursting activity (%SIB) of VTA DA at baseline and upon vmPFC stimulation. **(C)** PTSD group; **(D)** SEA group; **(E)** EA group; **(F)** Control group. PTSD and SEA rats exhibiting no differences in %SIB between eight baseline and vmPFC stimulation, whereas %SIB rose relative to baseline upon vmPFC stimulation in EA and control rats. *P < 0.05, vs. baseline.

Ventromedial prefrontal cortex stimulation was sufficient to evoke short- and long-latency excitatory and inhibitory responses in VTA DA neurons (Figures 7A,B,F). Of the 55 neurons analyzed in this study following vmPFC stimulation, 7 (12.7%) exhibited long-latency excitation, 24 (4.37%) exhibited short-latency excitation, 20 (36.3%) exhibited inhibition, and 4 (7.3%) exhibited no response. These results are in line with those of prior physiological studies assessing the impact of vmPFC stimulation on evoked DA neuron responses (Moorman and Aston-Jones, 2010). Chi-squared tests revealed no significant differences with respect to the types of VTA DA neuron responses in the four treatment groups (*P* > 0.05), nor were there any changes in mean firing rate over the course of vmPFC stimulation (Figures 7C,D). There were significant differences in bursting activity (%SIB) during ventral mPFC stimulation among treatment groups, with PTSD and SEA rats exhibiting no differences in %SIB between baseline and vmPFC stimulation (Figures 7A,B), whereas %SIB rose relative to baseline upon ventral mPFC stimulation in EA and control rats (*P < 0.05, Figure 7E).

## Discussion

Herein, we determined that EA treatment was sufficient to alleviate PTSD-associated anxiety-like behaviors in MSPS model rats. Immunofluorescent staining revealed that these anxiety-like behaviors were correlated with reduced vmPFC c-Fos expression, whereas EA treatment enhanced c-Fos levels in this region, consistent with functional vmPFC activation. Transient vmPFC inactivation ablated the ability of EA to alleviate anxiety-like behaviors. While PTSD model rats did not exhibit any change in VTA DA neuron %SIB upon vmPFC stimulation relative to baseline, such stimulation did increase the bursting activity of these neurons in rats in the EA treatment group. Together, our findings suggest that PTSD disrupts vmPFC activation and input into the VTA, potentially impacting the ability of the vmPFC to facilitate cognitive control of anxiety and thus giving rise to anxiety-like behaviors. EA-based therapy may thus be an effective treatment for PTSD owing to its ability to remediate pathological changes in the vmPFC and its inputs into the VTA.

The design of effective EA protocols is essential in order to effectively treat PTSD, with variables such as the choice of acupoints and the timing of treatment being of particular importance. Zhou et al. (2019) previously demonstrated the ability of EA pretreatment to prevent SPS-mediated induction of behaviors associated with anxiety. We and others have also previously shown that EA can similarly suppress anxiety-like behaviors following PTSD modeling (Liu et al., 2019). In this article, we found that a 14-day EA treatment period was sufficient to reduce the incidence of such behaviors, in line with prior results (Oh et al., 2018). These findings suggest that EA can benefit PTSD at a range of different stages during its development. Consistent with past reports (Zhou et al., 2019), we determined that low-frequency EA was able to suppress anxiety-like behaviors, suggesting that low-frequency stimulation may be more beneficial than high-frequency stimulation as a means of alleviating affective emotional and psychological states. Of course, this issue would need to be further clarified. According to traditional Chinese medicinal theory, the body contains over 300 acupoints, each of which is associated with its own therapeutic effects. The GV20, HT8, and HT7 acupoints are commonly selected when treating psychological or emotional disorders such as anxiety and depression (Oh et al., 2018; Zhou et al., 2019). In this study, we determined that EA conducted at the ST36 acupoints was sufficient to reduce the severity of anxiety-like behaviors in PTSD model rats. This acupoint is frequently used to treat pain, addiction, and disorders of the digestive system. These results suggest that this acupoint may also have value in the treatment of depression and anxiety. Additional study of synergistic combinations of ST36 and other acupoints may guide acupuncturists in the effective treatment of PTSD.

Our results suggested that EA can improve anxiety-like behaviors in PTSD model animals through mechanisms associated with the vmPFC. The mPFC is generally separated into the dorsolateral (dlPFC) and ventromedial regions (Koenigs and Grafman, 2009), with the dlPFC receiving sensory cortex input and being densely interconnected with premotor areas, frontal eye fields, and the lateral parietal cortex (Barbas, 2000), whereas the vmPFC protections are primarily associated with the amygdala, hypothalamus, and periaqueductal gray matter. The dlPFC is associated with executive and cognitive functions, while the vmPFC has been linked to the top-down regulation of emotional processes, attention, and executive functions. PTSD patients exhibit structural and functional vmPFC abnormalities that may contribute to fear and anxiety responses (Yi et al., 2011; Calhoon and Tye, 2015). Indeed, individuals suffering from PTSD associated with interpersonal violence exhibited reduced vmPFC activity in response to emotional scenes in an fMRI study relative to controls. The observed reductions in Fos-positive neurons in the vmPFC of PTSD model rats in this study were in line with those observed in prior analyses (Yu et al., 2015; Pati et al., 2018). Behavioral analyses have suggested that the vmPFC contributes to anxiety-like behaviors, and acute pharmacogenomic vmPFC excitatory neuron activation can markedly decrease the incidence of these behaviors (Pati et al., 2018; Salvi et al., 2019). Similarly, vmPFC deep brain stimulation (DBS) can decrease anxiety-like behavior incidence in a model of PTSD (Reznikov et al., 2018). Acupuncture analgesia has recently been linked to cortical modulation (Oh et al., 2018). The anterior cingulate cortex, for example, is critically linked to the efficacy of EA in the treatment of formalin-induced inflammatory pain model rats (Yi et al., 2011). Our lab has previously demonstrated that acupuncture can influence vmPFC neuron firing activity (Zhang et al., 2017). Herein, we additionally determined that EA significantly enhanced vmPFC c-Fos expression in PTSD model rats, while transient vmPFC inactivation was sufficient to reverse the beneficial effects of EA on anxiety-like behaviors in these animals. These data suggest that EA may represent a viable treatment alternative to DBS as a means of activating the vmPFC to treat symptoms associated with PTSD.

Dopamine neuron activation within the VTA is crucial as a means of preventing generalized anxiety (Zweifel et al., 2011; DeGroot et al., 2020), highlighting the potential value of enhancing DA neurotransmission in order to treat PTSD. In a prior study, for example, SPS model mice were treated with DA D2/D3 receptor agonists and exhibited the attenuation of PTSD-like symptoms (Malikowska-Racia et al., 2019). DA neuronal bursting results in significantly increased DA release, suggesting that this activity pattern is integral to the mesocorticolimbic DA system. Glutamatergic mPFC input to the VTA is also required for DA neuron burst activity (Murase et al., 1993; Tong et al., 1996), with this effect being dependent on NMDA receptor activation (Svensson, 2000). vmPFC stimulation activates VTA neurons (Gariano and Groves, 1988; Massi et al., 2008) and releases DA (Taber et al., 1995; You et al., 1998). Herein, we determined that PTSD model rats exhibited significantly decreased VTA DA neuron burst activity upon stimulation of the vmPFC, consistent with the disruption of mPFC inputs to the VTA as a consequence of this pathological condition. The anxiety-like behaviors associated with PTSD may thus be attributable to the abnormal functioning of this vmPFC-VTA neural circuit. A 14-day consecutive EA treatment regimen was sufficient to reverse these PTSD-associated reductions in VTA DA neuron burst activity, suggesting that the beneficial effects of EA on anxiety-like behaviors in this pathological context may be attributable to the impact of this therapeutic approach on VTA inputs from the vmPFC.

Certain factors must be considered when interpreting the results of our study. First, we utilized pentobarbital to anesthetize rats in our MSPS modeling approach, rather than ether, which is used for standard SPS modeling of anesthesia stress. We made this substitution owing to the fact that ether is both explosive and known to be toxic in humans and other mammals. In prior studies, no differences in gene expression, hematological findings, or biochemical parameters were observed when comparing treatments with ether, pentobarbital, and isoflurane (Nakatsu et al., 2017). Some researchers have reported that pentobarbital injection can induce additional stress that can interfere with cortisol levels in the plasma (Wu et al., 2015), and acute pentobarbital administration in rats is associated with the impairment of spatial learning, memory, and hippocampal long-term potentiation (Wang et al., 2015). In line with these reports, our PTSD model rats exhibited a range of anxiety-like behaviors. In addition to the differences in OFT and EPM performance detailed above, these animals exhibited reductions in body weight and climbing frequency as well as elevated corticosterone levels relative to control rats (supplementary data). Second, studies of SPS model animals and humans with PTSD have reported reductions in vmPFC activity. We found that EA treatment was associated with increased expression of c-Fos within the vmPFC, while transient vmPFC inactivation in our PTSD model rats was sufficient to ablate the effects of EA treatment on anxiety-like behaviors. These findings suggest that the vmPFC is thus implicated in the impact of EA on PTSD-related anxiety. Neurons within the vmPFC are primarily classified as putative pyramidal neurons and interneurons (85 and 15%, respectively) (Homayoun and Moghaddam, 2007; Sotres-Bayon et al., 2012). SPS model rodents have been shown to exhibit reduced mPFC glutamate levels (Knox et al., 2010; Lim et al., 2017; Piggott et al., 2019), indicating that vmPFC glutamate levels may mediate the effects of EA on the anxiety-like behaviors of PTSD model animals, although further research will be necessary to confirm this possibility. Single-unit extracellular recordings from rodents have additionally provided critical insights into the computational roles of DA neuron firing (Schultz, 2016). It is important to note that these recordings were made while rats were under the effects of isoflurane-induced anesthesia, which is likely to impact baseline activity. However, it should not qualitatively impact the effects of pathway activation (Zimmerman and Grace, 2016). In addition, the stability of brain states under controlled anesthesia conditions represents an experimental advantage, given that it enabled the unbiased identification of DA neuron firing. Our results thus provide direct insight into candidate mechanisms that can be tested in conscious animals in future studies. The DA neurons in the present study were identified based upon basal firing rate and waveform shape. As our study was focused on a small subset of DA neurons, additional electrophysiological analyses are necessary to validate and expand on our findings. Even so, our data offer a novel and robust foundation for future research.

## Data Availability Statement

The raw data supporting the conclusions of this article will be made available by the authors, without undue reservation.

## Ethics Statement

The animal study was reviewed and approved by Shanghai University of Traditional Chinese Medicine Animal Care and Use Committee.

## Author Contributions

YH, XS, BY, and SL conceived the study design. YH, LL, MC, and CW contributed to perform research. YH, LL, HM, XQ, and SL helped to draft and revise the manuscript. YH, LL, MC and CW, and SL analyzed the data. All authors participated in writing the manuscript and all have read and approved the final manuscript.

## Conflict of Interest

The authors declare that the research was conducted in the absence of any commercial or financial relationships that could be construed as a potential conflict of interest.
